# Neuroprotective effects of gemfibrozil in neurological disorders: Focus on inflammation and molecular mechanisms

**DOI:** 10.1111/cns.14473

**Published:** 2023-10-30

**Authors:** Mehraveh Sadeghi Ivraghi, Mohammad Yasin Zamanian, Reena Gupta, Harun Achmad, Hashem O. Alsaab, Ahmed Hjazi, Rosario Mireya Romero‐Parra, Enas R. Alwaily, Beneen M. Hussien, Elham Hakimizadeh

**Affiliations:** ^1^ School of Medicine Qazvin University of Medical Sciences Qazvin Iran; ^2^ Neurophysiology Research Center Hamadan University of Medical Sciences Hamadan Iran; ^3^ Department of Pharmacology and Toxicology, School of Pharmacy Hamadan University of Medical Sciences Hamadan Iran; ^4^ Institute of Pharmaceutical Research, GLA University Mathura India; ^5^ Department of Pediatric Dentistry, Faculty of Dentistry Hasanuddin University Makassar Indonesia; ^6^ Pharmaceutics and Pharmaceutical Technology Taif University Taif Saudi Arabia; ^7^ Department of Medical Laboratory Sciences College of Applied Medical Sciences, Prince Sattam bin Abdulaziz University Al‐Kharj Saudi Arabia; ^8^ Universidad Continental Lima Peru; ^9^ Microbiology Research Group College of Pharmacy, Al‐Ayen University Thi‐Qar Iraq; ^10^ Medical Laboratory Technology Department College of Medical Technology, The Islamic University Najaf Iraq; ^11^ Physiology‐Pharmacology Research Center Research Institute of Basic Medical Sciences, Rafsanjan University of Medical Sciences Rafsanjan Iran

**Keywords:** gemfibrozil, neuroinflammation, neurological disorders, neuroprotective, PPAR‐α

## Abstract

**Background:**

Gemfibrozil (Gem) is a drug that has been shown to activate PPAR‐α, a nuclear receptor that plays a key role in regulating lipid metabolism. Gem is used to lower the levels of triglycerides and reduce the risk of coronary heart disease in patients. Experimental studies in vitro and in vivo have shown that Gem can prevent or slow the progression of neurological disorders (NDs), including cerebral ischemia (CI), Alzheimer's disease (AD), Parkinson's disease (PD), and multiple sclerosis (MS). Neuroinflammation is known to play a significant role in these disorders.

**Method:**

The literature review for this study was conducted by searching Scopus, Science Direct, PubMed, and Google Scholar databases.

**Result:**

The results of this study show that Gem has neuroprotective effects through several cellular and molecular mechanisms such as: (1) Gem has the ability to upregulate pro‐survival factors (PGC‐1α and TFAM), promoting the survival and function of mitochondria in the brain, (2) Gem strongly inhibits the activation of NF‐κB, AP‐1, and C/EBPβ in cytokine‐stimulated astroglial cells, which are known to increase the expression of iNOS and the production of NO in response to proinflammatory cytokines, (3) Gem protects dopamine neurons in the MPTP mouse model of PD by increasing the expression of PPARα, which in turn stimulates the production of GDNF in astrocytes, (4) Gem reduces amyloid plaque pathology, reduces the activity of glial cells, and improves memory, (5) Gem increases myelin genes expression (MBP and CNPase) via PPAR‐β, and (6) Gem increases hippocampal BDNF to counteract depression.

**Conclusion:**

According to the study, Gem was investigated for its potential therapeutic effect in NDs. Further research is needed to fully understand the therapeutic potential of Gem in NDs.

## Graphical abstract

Neuroprotective effects of Gem on NDs. Gem exhibits neuroprotective properties by reducing inflammation, oxidative stress, and apoptosis in the brain. The mechanism of action involves modulating lipid metabolism, upregulating antioxidant enzymes, and inhibiting proinflammatory cytokines. Preclinical studies have shown that Gem treatment protects neurons, promotes neurite outgrowth, and improves cognitive function in animal models of neurodegenerative diseases like Alzheimer's and Parkinson's.

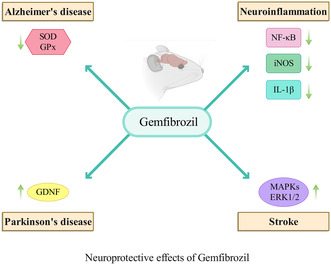

## 1 INTRODUCTION

Neurological disorders (NDs) are clinically characterized as pathologies that influence the cerebrum and the nerves that comprise the central and peripheral neural networks.^1^, ^2^ These diseases encompass various ailments: Parkinson's disease (PD), migraine headache, Alzheimer's disease (AD), multiple sclerosis, seizure disorder, as well as cerebrovascular accidents.^3^

NDs can cause slow and progressive deformations, leading to the loss of neurons and synapses. “Protein misfolding” diseases or proteinopathies are now the terms best describing the essential etiologies of NDs and defining these conditions.^4^, ^5^

The pathogenesis of NDs is initiated by several mechanisms, including the dynamics of protein misfolding, dysfunction of the proteasome, aggregation, defective degradation, oxidative stress (OS), formation of free radicals, mitochondrial dysfunction, DNA damage, disruption of axonal transport, neuroinflammatory or neuroimmunological procedures and changes in neurohumoral regulation.^6^, ^7^

Globally, cerebrovascular accident (CVA) burdens health systems with heavy morbidity and mortality and can be categorized into two types: ischemic and hemorrhagic.^8^ Ischemic CVA, accounting for approximately 80% of all CVA cases, is a cerebrovascular pathology resulting from stenosis or occlusion of cerebral arteries, leading to disruption of cerebral blood flow and ischemic necrosis or encephalomalacia of specific brain tissue.^9^ As a result of ischemia and hypoxia in brain tissues, the normal blood flow to neurons is disrupted in patients with ischemic CVA, leading to neuronal demise.^10^ Cerebral ischemia–reperfusion damage, inflammatory reactions, and excessive activation of microglia are critical mechanisms contributing to neuronal death in ischemic CVA.^11^ In contrast, AD is a progressive ND with a delayed commencement that typically manifests as amnestic cognitive deterioration, particularly impacting patients' activities of daily living (ADLs) and social interaction skills.^12^, ^13^ It possesses a considerable prevalence among individuals above the age of 65.^14^

AD is distinguished by the buildup of amyloid‐β (Aβ) plaques and the assemblage of Tau neurofibrillary tangles (NFTs) within the cerebrum.^15^ The build‐up of Aβ can lead to the formation of NFTs and the resulting neurodegeneration.^16^ Despite efforts to clear accumulated soluble and insoluble amyloid‐β plaques, clinical outcomes have not been promising.^17^ PD, an advancing neurodegenerative ailment affecting the central neural network, is common among individuals in middle age and the elderly.^18^, ^19^ The primary pathological characteristics of this condition encompass the dopaminergic neuron deterioration and demise taking place in the substantia nigra accompanied by the buildup of α‐synuclein in brainstem neurons, resulting in the formation of Lewy bodies.^20^ OS, mitochondrial malfunction, and inflammatory reactions all take part in the initiation and advancement of PD.^19^

The intrinsic properties of NDs make their management difficult and their prognosis unfavorable.^21^ Alteplase is the preferred therapy for acute ischemic CVA, but its limited therapeutic window and potential for lethal hemorrhage severely restrict its clinical application.^22^ Other agents, such as cholinesterase inhibitors, memantine hydrochloride, and levodopa, may offer some symptomatic relief for patients with NDs, but their therapeutic effects are suboptimal.^23^ As a result, there is a pressing need to develop neuroprotective medications that are both highly efficacious and have low toxicity.

Fibrates, a class of lipid‐lowering agents, have recently garnered attention for their prospective neuroprotective properties against a variety of cerebral disorders.^24^ As agonists of peroxisome proliferator‐activated receptor‐α (PPARα), fibrates can produce preemptive as well as immediate neuroprotective impacts via cerebral or vascular mechanisms.^25^ Gemfibrozil (Gem; PPARα agonist), a fibrate marketed under the brand name Lopid, has been safely used in humans to treat hypertriglyceridemia since its endorsement by the Food and Drug Administration (FDA) in 1982.^26^

The widespread production of PPARα in various tissues suggests that Gem may have effects on multiple areas of the body.^27^

During global cerebral ischemia/reperfusion (I/R) it has been shown that Gem has an impact on the brain by having effects on specific areas of its brain. In this study, it was observed that Gem pretreatment in female rats resulted in the modulation of inflammatory factors, including tumor necrosis factor‐a (TNF‐a), nuclear factor kappa B (NF‐κB), and cyclooxygenase‐2 (COX‐2).^24^

Neuroinflammatory diseases are a group of disorders that involve inflammation in the central nervous system (CNS).^28^ These diseases can be caused by a variety of factors, including infections, autoimmune reactions, and traumatic brain injury.^29^ Examples of neuroinflammatory diseases include multiple sclerosis, AD, and PD.^30^

Gem can suppress the expression of pro‐inflammatory molecules in human primary microglia via activation of PPAR‐α. The studies suggest that Gem could have potential therapeutic value in several neuroinflammatory and NDs since it regulates both microglial activation and inflammatory gene expression in a PPAR‐β dependent manner, suggesting that Gem may represent a promising treatment for these conditions.^31^

In a rodent AD model, Gem reduced the load of amyloid deposits in the hippocampus and cortex, decreased microgliosis and astrogliosis linked to plaque formation, and enhanced rodent spatial memory and learning.^32^

Research on rodents with PD suggests that taking Gem may provide a protective effect for dopaminergic neurons due to its impact on lipid profiles. A study conducted on mice found that oral administration of Gem protected the substantia nigra pars compacta dopaminergic neurons, as well as the striatal TH fibers of MPTP‐injured mice of both genders. As a result, Gem improved the locomotor abilities of mice intoxicated with MPTP by regulating the neurotransmitters in the striatum.^33^

Interleukin‐1 receptor antagonist (IL‐1Ra) is an important molecule in attenuating inflammation as it binds to the same receptor as interleukin‐1 beta (IL‐1β), a proinflammatory cytokine, and inhibits proinflammatory cell signaling.^34^ Corbett et al. reported that Gem can upregulate the expression of IL‐1Ra in mouse cortical neurons.^35^ Gem‐induced upregulation of IL‐1Ra is mediated by the activation of the PI3‐K – Akt – CREB.^35^ This upregulation of IL‐1Ra by Gem is suggested to enhance the defense mechanism of cortical neurons against neuroinflammatory and neurodegenerative disorders.

Considering the insufficient body of evidence with regard to the neuroprotective effects of Gem, the present study has looked into its probable therapeutic effect in NDs mainly concentrating on cellular and molecular signaling pathways.

## 2 OVERVIEW OF GEMFIBROZIL

Gem, with the chemical formula C_15_H_22_O_3_, was initially demonstrated to lower lipid levels in animals in the 1960s and was subsequently approved by the FDA in 1976 to be utilized to decrease serum lipids in animals.^36^, ^37^ It has been shown to decrease levels of triglycerides, very low‐density lipoprotein, and low‐density lipoprotein while boosting high‐density lipoprotein.^38^ Gem promotes the nuclear receptor PPAR‐α, which is crucial for controlling lipid metabolism.^39^

Gem is a medication utilized to manage hypertriglyceridemia caused by retinoids.^40^ It is more effective than pravastatin, another lipid‐lowering medication, in reducing triglycerides and increasing HDL cholesterol.^41^ This drug is primarily used to treat individuals with Type IV and Type V hyperlipidemia who are at risk of developing cardiovascular ailments, coronary vascular issues, and other lipid‐associated conditions.^38^, ^42^

Gem is ingested orally in tablet form, with a recommended dosage of approximately 20 mg/kg.^43^ Each tablet contains 600 mg of Gem and is taken with water.^44^ Plasma concentrations of Gem are measured at various time points following administration, with peak concentration occurring at 2 h and decreasing by 50% after 4 h. Renal clearance of Gem takes place after roughly 1.5 h, with up to 50% of the drug being eliminated as conjugates.^45^ It has been found that approximately 70% of these conjugates are eliminated through urination.^46^ A separate investigation proposed that the glucuronidation process of Gem is essential in reducing its effectiveness, with the conjugated variant of the medication, gemfibrozil 1‐O‐β‐glucuronide, functioning as a metabolite‐dependent suppressor of CYP2C8.^47^, ^48^

Gem is a medication that can interact with other drugs and cause side effects.^49^ When taken with statins, such as atorvastatin, lovastatin, and simvastatin, it can increase the risk of muscle toxicity.^50^ It can also increase the anticoagulant effect of warfarin, which may heighten the likelihood of hemorrhaging.^51^ Furthermore, Gem can raise the concentration of repaglinide and cyclosporine, which can increase the risk of hypoglycemia and kidney damage, respectively.^52^

Some patients may experience gastrointestinal symptoms such as dyspepsia, nausea, vomiting, cholelithiasis, and gallstones while taking Gem.^53^ Other side effects include dizziness, vertigo, myopathy, rhabdomyolysis, and allergic reactions such as angioedema, urticaria, and rash.^53^, ^54^ Taking Gem with food can help reduce gastrointestinal symptoms.^55^

## 3 NEUROPROTECTIVE EFFECTS OF GEMFIBROZIL ON NEUROLOGICAL DISORDERS

### 3.1 Cerebral ischemia (CI)

Cerebral ischemia is a medical condition in which there is a lack of blood flow to the brain, leading to a shortage of oxygen and glucose needed for cellular metabolism.^56^ This can result in damage or death of brain cells and can lead to various neurological symptoms and disorders, including stroke.^57^

NRF‐1 is a transcriptional regulator that controls the manifestation of nuclear genetic material involved in mitochondrial biogenesis, oxidative phosphorylation, and the replication and transcription of mitochondrial DNA.^58^, ^59^ The brain requires it to maintain mitochondrial function and energy metabolism.^60^ NRF‐1 interacts with PGC‐1α and other transcription factors to regulate mitochondrial biogenesis.^61^ Dysfunction of NRF‐1 has been linked to several NDs.^62^, ^63^

TFAM is found to partake in regulating the replication and transcription of mitochondrial DNA.^64^ It interacts with mitochondrial DNA to help it condense into nucleoids and controls the transcription of genes that code for the components of the respiratory complex.^65^ TFAM is considered a pro‐survival factor for mitochondria and is involved in the signaling pathway for mitochondrial biogenesis.^59^

JNK is a subtype of mitogen‐activated protein kinase that is pivotal in moderating various cellular pathways, encompassing cell proliferation, differentiation, survival, and apoptosis.^66^, ^67^ The JNK pathway is activated in response to a wide variety of extracellular stimuli, including stress, cytokines, and growth factors.^68^ Once activated, JNK phosphorylates its downstream targets, including transcription factors and other kinases, leading to changes in gene expression and cellular responses.^69^ JNK is present in the process of several diseases; that is cancer, neurodegenerative disorders, and inflammatory conditions.^70^, ^71^

ERK1/2 is classified as a kind of protein kinases that are pivotal in moderating numerous cellular processes, encompassing cell proliferation, differentiation, survival, and apoptosis.^72^, ^73^ ERK1/2 is involved in mitogen‐activated protein kinase signaling, an important pathway activated by growth factors, cytokines, and stress.^74^ ERK1/2 is involved in regulating mitochondrial biogenesis and cell survival, with its activation promoting cell survival and proliferation.^75^ Dysregulation of ERK1/2 has been linked to several NDs, comprising AD, PD, and cerebrovascular accidents.^76^, ^77^, ^78^

Mohagheghi et al. studied the global cerebral ischemia–reperfusion (I/R) injury and described a neurodegeneration resistance in male rats, while met‐estrous females exhibited extensive damage in the hippocampal CA1 region. However, pretreatment with Gem had a sexually differentiated influence and provided a neuroprotection effect in met‐estrous females but caused neurodegeneration in males. They found that Gem had a stimulating effect on the expression of NRF‐1 and TFAM specifically within the hippocampus of met‐estrous females in the mitochondrial biogenesis‐signaling pathway. Additionally, Gem modulated apoptotic cell death pathways and upstream ERK1/2, MAPKs and JNK in a sexually dimorphic manner. The study suggests that the sex‐dependent effect of Gem pretreatment is due to estrogen‐ and testosterone‐dependent mechanisms.^24^

Inflammation is a key pathophysiological mechanism involved in CI.^79^ Inflammatory factors such as NF‐κB, TNF‐α, and COX‐2 have been shown to be conceivably stimulated subsequent to cerebral trauma, promoting neuroinflammation and neuronal deterioration.^80^, ^81^ In cases of CI, NF‐κB can contribute to neuronal cellular demise, mainly if the ischemia is acute and results in irreversible brain damage.^82^ As such, inflammation can exacerbate the damage caused by CI.

Nrf2 transcription factor governs a broad range of antioxidant genes, which work together to eliminate ROS through a series of enzymatic reactions.^19^ Nrf2 specifically targets genes encoding antioxidant enzymes that contain an antioxidant response component (ARE) within their promoters, including HO‐1, GPx, and NQO‐1.^7^, ^83^ These enzymes help support redox balance as well as affect the inflammatory reaction. As such, Nrf2 is crucial in safeguarding cells against OS and inflammation.

According to a study by Mohagheghi et al., pretreatment with Gem influences the antioxidant defense system and inflammatory pathways in female rats, leading to neuroprotection. The findings suggest that Gem pretreatment has varying impacts on male and female rodent models of I/R injury. While males were found to be resistant to 10 min of global CI, metestrous females exhibited extensive neurodegeneration in the hippocampus CA1 area in identical circumstances. Gem was shown to have neuroprotective effects in females but was neurotoxic in males. The inflammatory pathway was more impacted by Gem than the Nrf‐2 signaling pathway. It is necessary to conduct additional research in order to establish a causal relationship between these observations.^84^

MCAO refers to a type of stroke that results from an obstruction, typically a blood clot, in the middle cerebral artery of the brain.^85^ Depending on how long the obstruction goes on and with how severity, this can cause brain damage and neurological impairments.^86^ MCAO is frequently employed as a model for investigating ischemic stroke in animals.^85^

Guo et al. showed that treatment with Gem, a PPAR‐alpha agonist, improved functional outcomes and led to infarct zone reduction in mice after permanent MCAO. The study measured neurological deficit, pole test, and accelerated rotarod test as functional outcomes and found that Gem treatment improved performance on these tests compared to control mice. The study also analyzed protein and mRNA levels, as well as total cholesterol and triglyceride levels, and found that Gem treatment had beneficial effects on these measures as well. Overall, the study suggests that Gem could be promising as a neuroprotective agent in stroke and other neurodegenerative diseases.^87^

### 3.2 Neuroinflammation

Neuroinflammation refers to inflammation that occurs within the neural network, specifically in the brain and spinal cord.^88^ It is marked by the activation of glial cells, including microglia and astrocytes, which release proinflammatory cytokines and chemokines.^89^ Neuroinflammation can arise from a multitude of causes, including infection, injury, autoimmune disorders, and neurodegenerative diseases.^90^ Several NDs, including AD, PD, multiple sclerosis, and stroke, have been shown to be influenced by persistent neuroinflammation.^91^

Microglia are a type of glial cells that functions as the primary macrophage in the CNS.^92^ They possess a pivotal role in maintaining homeostasis in the CNS by removing damaged cells and debris, as well as by responding to infections and injuries.^93^ In addition, microglia play a role in the pruning of synaptic connections during development and in the regulation of neurogenesis.^94^ When activated, microglia undergo morphological changes and produce proinflammatory cytokines, chemokines, and ROS that can contribute to neuroinflammation.^95^ Abnormal activation of microglia was shown to be related to the development of several NDs, encompassing AD, PD, multiple sclerosis, and stroke.^96^, ^97^

CD11b, also known as macrophage‐1 antigen, is a cell surface glycoprotein that is also referred to as integrin alpha M.^98^ It combines with CD18 (integrin beta 2) to form the Mac‐1 integrin heterodimer, expressed on the external membrane of various immune cells, including microglia.^99^, ^100^ CD11b/CD18 is involved in cell adhesion, migration, and phagocytosis.^101^ Elevated expression of CD11b/CD18 has been observed in activated microglia in various neuroinflammatory and neurodegenerative conditions such as AD and multiple sclerosis.^102^, ^103^ As such, CD11b/CD18 is suggested to be a promising treatment option for these conditions.

Research in human microglia suggests that bacterial lipopolysaccharides trigger the increased production of numerous pro‐inflammatory substances and CD11b expression on the surface of microglial cells.^31^, ^104^

Jana and Pahan discovered that Gem, a drug used to lower lipid levels, hinders the stimulation of primary human microglia through PPAR‐ β. Specifically, as a result of using Gem, the proinflammatory molecules and CD11b were expressed to a lesser degree in primary human microglia stimulated by LPS. The study also revealed that clofibrate, another hypolipidemic drug that leads to activation of PPAR‐α, was also able to impede the expression of proinflammatory molecules in human microglia, although it was less effective than Gem. Moreover, the study demonstrated that Gem reduced the level of iNOS and proinflammatory cytokines that were induced by LPS in human microglia in a dose‐dependent manner. Finally, the study showed that PPAR‐β in human primary microglia and astrocytes was manifested to a higher degree as a result of Gem.^31^

Nitric oxide (NO) is a diffusible gas with a short half‐life that serves as a signaling and effector particle in living systems.^105^ It participates in a variety of physiological processes such as vasodilation, neurotransmission, and immune response.^106^ However, excessive NO production is associated with CNS disorders, such as inflammatory, degenerative, traumatic, and infectious.^107^, ^108^

iNOS is the facilitating enzyme for the generation of NO in response to a spectrum of stimuli, such as proinflammatory cytokines, bacterial endotoxins, and OS.^109^, ^110^ The overproduction of NO by iNOS has been associated with several pathological conditions, including inflammation, neurodegeneration, and cancer.^111^, ^112^

Astrocytes are a subcategory of glial cells in the CNS that provide support and protection for neurons.^113^ They play essential roles in sustaining the structural integrity of the brain, regulating blood flow, and modulating synaptic activity.^114^, ^115^ Astrocytes also participate in various physiological processes, including neurotransmitter recycling, ion homeostasis, and immune response.^116^ There is evidence that dysfunction of astrocytes can contribute to a number of neurodegenerative diseases, including AD, PD, and multiple sclerosis.^117^

Pahan et al. discovered that Gem inhibited human astrocyte nitric oxide production and iNOS expression. The authors also observed that Gem effectively reduced iNOS expression in human U373MG astroglial cells and primary astrocytes, indicating that Gem is effective at reducing iNOS expression. Furthermore, IL‐1β and IFN‐γ together significantly increased the production of NO, but this effect was inhibited by Gem. Gem was also found to inhibit the activation of NF‐κB, AP‐1, and C/EBPβ in cytokine‐stimulated astroglial cells, which are known to induce the expression of iNOS and the production of NO in response to proinflammatory cytokines. Overall, the findings suggest that Gem may have potential therapeutic uses for conditions associated with excessive nitric oxide production in the brain.^118^

IL‐1Ra is a protein that naturally occurs in the body and produces anti‐inflammatory effects by binding to the IL‐1R1 receptor in a competitive manner, which is the receptor for the proinflammatory cytokine (IL‐1β).^119^ While IL‐1β activates proinflammatory signaling pathways, IL‐1Ra inhibits proinflammatory cell signaling by attaching to the same receptor.^120^ As a result, increasing the expression of IL‐1Ra is believed to be important for reducing inflammation.^121^

Phosphatidylinositol 3‐Kinase (PI3‐K) is a bifunctional kinase that has the ability to transmit signals from various signaling pathways that are involved in various biological processes.^122^ The PI3‐K signaling molecule regulates many biological processes, including mitogenesis, oxidative bursts, and cell survival.^123^

According to Corbett et al. Gem increased the expression of IL‐1Ra in fetal mouse cortical neurons (fMCNs). The authors also noted that Gem‐activated type IA p110α phosphatidylinositol 3‐kinase (PI3‐K) and Akt, which participate in cell survival and growth signaling pathways. The described observations propose that Gem possibly has therapeutic value in reducing chronic inflammation in neurodegenerative disorders.^35^

LINCL is an uncommon neurodegenerative disorder due to Cln2 gene mutation, resulting in a deficient or nonfunctional tripeptidyl peptidase 1 (TPP1) enzyme.^124^ LINCL typically manifests symptoms at 2–4 years of age, advances rapidly, and results in death between the ages of 8 and 10 due to a significant cell death in neurons and other cells.^125^ Several studies have determined that neuro‐inflammation and the activation of apoptosis processes contribute to neuronal damage in most NCL subtypes, including LINCL.^126^, ^127^

SOCS3 (suppressor of cytokine signaling 3) is a protein that regulates the immune response and inflammation in the body.^128^

According to Ghosh and his colleagues, treatment with Gem resulted in improved motor activity, reduced neuronal apoptosis, and increased anti‐inflammatory factors in the brains of mice with LINCL. Specifically, Gem administration increases SOCS3 and IL‐1Ra in both striatum and motor cortex in Cln2 (−/−) mice after 8 weeks. The increase in SOCS3 was evident in astrocytes, microglia, and other brain cells. These observations suggest that Gem may have potential therapeutic value for patients with LINCL.^126^

IκBα is a protein that plays a fundamental part in controlling the action of the transcription factor NF‐κB.^129^ This transcription factor is responsible for regulating many genes that are essential for the immune response, including those that regulate inflammation.^130^ IκBα works by binding to NF‐κB and preventing it from entering the cell nucleus, where it can activate the transcription of its target genes.^131^ When IκBα is broken down, NF‐κB is freed and can enter the nucleus to activate gene expression.^131^

According to research by Jana et al., gemfibrozil's anti‐inflammatory effect is partly due to the activation of PI3K. The drug was found to increase the expression of the anti‐inflammatory molecule IκBα in microglia through the PI3K pathway. By activating this pathway, Gem inhibits the activation of NF‐κB in microglia. This results in the retention of NF‐κB in the cytoplasm, preventing it from activating the transcription of genes that promote inflammation. These findings indicate that Gem could be a potential treatment for brain inflammation.^132^

Krüppel‐like factor 4 (KLF4) is a transcription factor that contains zinc fingers and is part of the KLF protein family. It takes part in controlling embryogenesis, cell proliferation, apoptosis, differentiation, tumor formation, and inflammation.^133^

According to research by Ghosh and Pahan, Gem increases the expression of SOCS3 in glial cells in a way that is dependent on both time and dosage. The study also found that Gem activates type IA phosphatidylinositol (PI) 3‐kinase and AKT and also proved that blocking PI 3‐kinase and AKT with chemical inhibitors prevented the Gem‐induced increase in SOCS3 expression.

Additionally, the study described that Gem activates KLF4 through the PI 3‐kinase‐AKT pathway and also showed that reducing KLF4 levels using siRNA prevented the Gem‐induced increase in SOCS3 expression. Overall, it is concluded from these findings that Gem probably has therapeutic effect for various neuroinflammatory and neurodegenerative diseases by increasing SOCS3 levels through the activation of KLF4 via the PI 3‐kinase pathway.^134^

### 3.3 Alzheimer's disease (AD)

Alzheimer's disease is a disorder with neurodegenerative nature that progressively weakens the brain, causing memory loss, cognitive decline, and changes in behavior.^135^ It is the main disease leading to dementia in older adults and is resulted from the buildup of beta‐amyloid plaques and tau protein tangles in the brain, which result in the death of brain cells and the shrinking of brain tissue.^136^ There is no precise etiology for AD, but it appears to be linked to a variety of factors including genetics, environment, and lifestyle.^137^ While no definite cure has been found, some treatments are proven to help manage symptoms and improve patients' quality of life.

Gem is a medication that has been used for over four decades to reduce triglyceride levels in the blood.^138^ There is evidence to suggest that high triglyceride levels may be a risk factor for AD.^139^ Triglycerides are fat molecules in the blood that the cells utilize for energy.^140^ However, at high levels, they participate in the formation of plaques in the brain, which are a characteristic feature of AD.^140^ Additionally, there is evidence to suggest that triglycerides may affect the release and breakdown of beta‐amyloid peptides, which are responsible for changes leading to AD.^140^ More research is in demand to properly display the relationship between triglycerides and AD.

SH‐SY5Y is a human neuroblastoma cell line that is commonly used as a model for studying neuronal function and neurodegenerative diseases such as AD.^141^ The origin of these cells is the bone marrow of a 4‐year‐old female with neuroblastoma and have been extensively characterized for their neuronal properties.^142^ They are widely used in research since they are able to transform into neurons and exhibit many of the same properties as primary neurons, making them a useful tool for studying neuronal function and disease mechanisms.^143^

PPARα is a nuclear receptor that takes part in regulating lipid metabolism and energy balance.^144^ Its main function is in tissues that are entangled with breaking down fatty acids, such as the liver, heart, and skeletal muscle.^145^, ^146^ PPARα is triggered by fatty acids and their byproducts, as well as by certain medications, including fibrates and thiazolidinediones.^147^ When activated, PPARα forms a complex with retinoid X receptor (RXR) and attaches to specific DNA sequences called PPREs in the promoter regions of target genes.^148^ This leads to the activation of these genes' transcription. PPARα regulates the expression of genes responsible for fatty acid uptake, transport, and oxidation, as well as ketone body synthesis and glucose metabolism.^149^

The T maze is a behavioral test used to evaluate the memory of mice.^150^ In this test, mice are trained for 2 days on a T‐shaped maze and are deprived of food beforehand to provoke them to seek the food placed on the right side of the maze as an incentive.^151^

The Barnes maze is another behavioral test used to assess spatial memory in rodents, particularly mice. It consists of a circular platform with multiple holes around its perimeter, one of which leads to an escape tunnel. During training, the mouse is put in the center of the maze and allowed to move until it finds the escape tunnel. The location of the escape tunnel remains constant throughout training. After training, the mouse is tested for its ability to remember where the escape tunnel is located. The time it takes for the mouse to look for the escape tunnel and the number of errors it makes are recorded as measures of spatial memory. The Barnes maze is often used in research on AD and other NDs that affect spatial memory.^32^, ^152^

Chandra and Pahan reported that Gem, a medication with a lipid‐lowering effect, can also lower amyloid plaque pathology and enhance memory in a mouse model of AD via PPARα. They used various tests, including the T maze and Barnes maze, to evaluate memory and the open field experiment to analyze locomotor function. They also used immunohistochemistry and densitometric analysis to measure amyloid plaque pathology. The results showed that Gem treatment reduced amyloid plaque pathology and improved memory in the mice.^32^

Hakimizadeh et al. found that Gem has potential benefits for brain function in aging mice. Specifically, the study found that Gem reduced anxiety‐like behavior, improved memory, and reduced OS in the brains of aging mice. They found that Gem prevented the decrease in antioxidant enzymatic activities and increase of malondialdehyde (MDA) levels in the brains of D‐galactose‐induced aging mice. These findings support the possibility of neuroprotective effects for Gem through reduction OS in the brain.^153^ These results indicate that Gem could be a potential treatment for aging‐related cognitive decay, including AD.

### 3.4 Parkinson's disease (PD)

Glial cell line‐derived neurotrophic factor (GDNF), is a protein that is essential for the survival and growth in dopaminergic neurons, which are the cells that are lost in PD.^154^ GDNF is produced by astrocytes, a type of brain cell, and acts as a trophic factor for dopaminergic neurons.^155^ A reduction in GDNF production in the brain has been revealed to contribute to the degeneration of nigrostriatal pathways in PD.^156^

The MPTP neurotoxin damages dopaminergic neurons in the brain, causing PD‐like symptoms.^157^ MPTP is often used to create animal models of PD for research purposes.^158^

Dopaminergic neurons are brain cells that produce dopamine, a neurotransmitter that plays a crucial role in regulating movement, incentive, and reward.^159^ These neurons are mainly found in the SNpc region of the brain, and their loss is a characteristic feature of PD.^160^ Dopaminergic neurons are important for the proper functioning of the basal ganglia, a group of brain structures involved in motor control and other functions.^161^

According to research by Gottschalk et al., Gem can protect dopaminergic neurons in a mouse model of PD through a PPARa‐dependent astrocytic GDNF signal. Gem was found to improve nigrostriatal pathology and motor deficits in mice treated with MPTP, a neurotoxin that causes Parkinson's‐like symptoms. The drug was also found to increase the expression of GDNF in astrocytes, which supports the survival of dopaminergic neurons. These discoveries propose the possibility of Gem being a potential treatment for PD. The study also used various tests to evaluate the effects of Gem on motor function and behavior in mice.^33^

### 3.5 Demyelinating diseases

Myelination is the process by which nerve fibers are coated with a fatty substance called myelin, which acts as an insulator and allows for faster and more efficient transmission of electrical signals along the nerve fibers.^162^ Myelin is produced by specialized cells called oligodendrocytes and Schwann cells in the CNS and peripheral nervous system (PNS) respectively.^163^ The process of myelination begins during fetal development and continues through adolescence, with some myelination occurring throughout adulthood.^164^ Myelination is essential for proper nervous system function, and disruptions in myelination can lead to a variety of NDs, including multiple sclerosis.^165^

Multiple sclerosis (MS) is a CNS disorder with an autoimmune nature, which affects the brain, spinal cord, and optic nerves.^166^ In MS, the immune system assaults the protective myelin sheath surrounding CNS neurons.^167^ This can cause a range of symptoms, including localized weakness, numbness, vision problems, and problematic coordination and imbalance.^168^ MS is a progressive disease, meaning that symptoms can worsen over time.^168^ Never a cure has been introduced but symptoms can be managed to some extent and progression of the disease can be slowed with some treatments.

According to research by Jana et al., Gem, a medication commonly used to reduce cholesterol levels, can increase the expression of myelin genes in human oligodendrocytes through PPAR‐α. Specifically, Gem was found to increase the expression of numerous myelin‐specific genes, such as MBP, CNPase and PLP in mixed glial cells, primary human oligodendrocytes and spinal cord organotypic cultures. These findings suggest that Gem has a myelinogenic property that could be beneficial for treating demyelinating disorders, for instance, multiple sclerosis.^169^

Oligodendrocytes are a variety of glial cell located in the CNS that produces myelin, a lipid‐rich substance that creates a shielding layer around nerve fibers.^162^ This sheath helps to insulate nerve fibers and allows for faster and more efficient transmission of electrical signals between neurons.^170^ Oligodendrocytes are essential for proper neuronal function, and abnormalities in myelin in the CNS are linked to a range of NDs.^171^

SREBF is a transcription factor that dictates gene expression related to cholesterol and fatty acid metabolism.^172^ When cellular cholesterol levels are low, SREBFs are activated and stimulate gene expression in cholesterol synthesis and uptake.^173^ SREBFs also play a role in regulating other cellular processes, including glucose metabolism and myelination.^174^, ^175^

According to research by Ashikawa et al., medications such as fenofibrate and Gem, which are used to treat high cholesterol levels, can promote myelination in zebrafish. The study suggests that the pro‐myelinating effects of these drugs occur through the activation of SREBFs. These findings provide evidence that activating SREBFs may be a potential therapeutic approach for stimulating myelination.^176^

Interferon‐gamma (IFN‐γ) is a type of cytokine that has a pivotal function in defending against infections caused by viruses, bacteria, and parasites.^177^ This cytokine is generated by activated T cells and natural killer (NK) cells, and it helps regulate the growth, differentiation, and activation of immune cells.^178^ Additionally, IFN‐γ possesses anti‐tumor properties and is utilized to treat certain types of cancer. IFN‐γ can promote Th1 immune responses and exacerbate disease symptoms in autoimmune diseases like multiple sclerosis.^179^, ^180^

GATA3 is a transcription factor pivotal in the development of T helper 2 (Th2) cells.^181^ This factor is found in activated CD4+ T cells and is responsible for generating Th2 cytokines such as IL‐4, IL‐5, and IL‐13.^182^ Furthermore, GATA3 helps regulate other immune cells, including eosinophils and mast cells.^182^ In autoimmune diseases like multiple sclerosis, this transcription factor is associated with promoting Th2 immune responses, which can improve disease symptoms.^183^

T‐bet, also known as T‐box expressed in T cells, is a transcription factor that has a pivotal function in the development of T helper 1 (Th1) cells.^184^ This factor is present in activated CD4+ T cells and is pivotal in generating the Th1 cytokine IFN‐γ.^185^ Additionally, T‐bet helps regulate other immune cells, encompassing NK cells and dendritic cells.^186^ In autoimmune diseases like multiple sclerosis, this transcription factor is associated with promoting Th1 immune responses, which can worsen disease symptoms.^187^

In their study, Dasgupta et al. discovered that Gem, a drug used to lower lipid levels, improves relapsing–remitting experimental autoimmune encephalomyelitis (RR‐EAE) independently of PPAR‐α. The drug was shown to alter the differentiation of myelin basic protein (MBP)‐primed T cells from Th1 to Th2 and decrease the production of the Th1 cytokine IFN‐γ. Additionally, Gem reduced the clinical symptoms of RR‐EAE. Additionally, the study demonstrated that the NO plays an important role in regulating T‐bet and GATA3 expression, and that Gem reduces EAE independently of PPAR‐α in both wild‐type and knockout mice.^188^

### 3.6 Major depressive disorder

Major depressive disorder is described as affecting an individual's mental well‐being by enduring emotions of melancholy, despair, and a lack of engagement in once pleasurable pursuits.^189^, ^190^ This condition can also cause physical symptoms such as exhaustion, changes in eating and sleeping habits, and difficulty focusing.^191^ It can have a significant impact on a person's daily life and may necessitate treatment such as therapy and medication.^192^

HAM‐D is a standardized questionnaire used to evaluate the severity of depression in patients.^193^ It consists of 21 items that assess the presence and intensity of depressive symptoms such as sadness, guilt, insomnia, and anxiety.^194^ Each item is scored on a scale of 0 to 2 or 0 to 4, with elevated scores signifying more intense manifestations.^195^ The cumulative score is between 0 and 63, with scores above 24 indicating severe depression. The HAM‐D score is frequently used in clinical trials and research studies to measure the effectiveness of treatments for depression.^195^

In their study, Zandifar et al. found that when taken at a dose of 300 mg per day, Gem can be an effective additional treatment for individuals afflicted with major depressive disorder. The research demonstrated a considerable reduction that was statistically significant in HAM‐D score in the Gem group compared to the placebo group during the first 2 weeks. Additionally, the rate of remission was considerably elevated in the Gem cohort in the 8 weeks.^196^

pCREB is a protein that is vital in controlling the manifestation of genes as a reaction to various stimuli, encompassing neurotransmitters and growth factors.^197^ It is a downstream signaling molecule of BDNF, and its phosphorylation is required for the activation of BDNF‐mediated signaling pathways.^198^ In relation to depression, reduced levels of pCREB are seen in the hippocampus, the part of the brain that regulates moods.^199^

In their investigation, Ni et al. uncovered that Gem exhibits antidepressant effects in mice and may hold promise as a novel antidepressant. The investigation utilized an array of tests to gauge the effects of Gem, including the forced swim test (FST), tail suspension test (TST), sucrose preference test, and chronic unpredictable mild stress (CUMS) model of depression. They determined that Gem facilitates mobility in the FST and TST, enhanced sucrose preference, and counteracted the effects of CUMS‐induced depression. Furthermore, they ascertained that Gem augmented the expression of BDNF and pCREB in the hippocampus, indicating that the BDNF system is pivotal in the antidepressant effects of Gem.^200^

Overall, these studies suggest that Gem may have various effects on the brain, including reducing anxiety, enhancing memory, and promoting the BDNF system. However, more research is needed to fully understand the drug's effects on the brain and its potential therapeutic uses. A summary of previous studies is provided in Table 1 of the current study, which gives an overview of previous studies.

**TABLE 1:** Studies consistent with the purpose of this study.

Authors	PPAR subtype	Dosage of Gem	Type of study/Model	Mechanisms
Mohagheghi et al.	PPAR‐α	30 mg/kg	Rat Models of Global Cerebral Ischemia–Reperfusion	Modulats apoptotic cell death pathways and upstream MAPKs, JNK and ERK1/2 in a sexually dimorphic manner
Guo et al.	PPAR‐α, PPAR‐β, and PPAR‐γ	30 mg/kg	Middle cerebral artery occlusion (MCAO) models in mice	Improves functional outcomes and led to infarct zone reduction in mice after permanent MCAO
Jana and Pahan	PPAR‐α, PPAR‐β, and PPAR‐γ	50–200 μm	Human microglia	Reduces LPS‐induced expression of iNOS and proinflammatory cytokines in human microglia in a dose‐dependent manner
Pahan et al.	PPAR‐α	150–200 μM	Human U373MG astroglial cells	Reduces the expression of iNOS in human U373MG astroglial cells
Corbett et al.	Not mentioned	25 μM	Human primary neurons	Activation of type IA p110α phosphatidylinositol 3‐kinase (PI3‐K) and Akt
Chandra and Pahan	PPAR‐α	7.5 mg/kg	Mouse model of Alzheimer's disease	Reduces amyloid plaque pathology and improved memory
Gottschalk et al.	PPAR‐α	7.5 mg/kg	Mouse model of Parkinson's disease	Protects dopaminergic neurons in a mouse model of PD through a PPARa‐dependent astrocytic GDNF pathway
Jana et al.	PPAR‐β	25 and 50 μM	Human oligodendrocytes	Increases the expression of numerous myelin‐specific genes, such as myelin basic protein, myelin oligodendrocyte glycoprotein, 2′,3′‐cyclic‐nucleotide 3′‐phosphodiesterase, and proteolipid protein (PLP), in primary human oligodendrocytes
Dasgupta et al.	PPAR‐α	300 mg/kg	Experimental allergic encephalomyelitis (EAE) model in female SJL/J mice	Reduces the clinical symptoms of RR‐EAE, prevents activated myelin‐specific T cells and/or inflammatory cells from entering the CNS parenchyma
Zandifar et al.	Not mentioned	300 mg daily	Clinical Trial	Gem can be an effective additional treatment for individuals afflicted with major depressive disorder

In summary, Gem passes the BBB and enters the central nervous system. Gem, has shown potential as a therapeutic approach for various cerebral disorders, including cerebral injury, ischemic stroke, and global cerebral IR injury. It has been reported to have antioxidant and anti‐inflammatory effects, as well as the ability to stimulate antioxidant enzyme expression. Gem has been shown to protect against cerebral injury through its antioxidant and anti‐inflammatory mechanisms. However, it is important to note that the neuroprotective/neurodegenerative effects of Gem may vary depending on the specific condition and timing of administration.

Gem has been shown to have a positive effect on preventing the development of neurodegenerative diseases, including AD and PD in experimental studies.

However, more research and clinical studies are needed to fully understand the potential therapeutic benefits and safety profile of Gem in different NDs.

## 4 CONCLUSION

**FIGURE 1:**
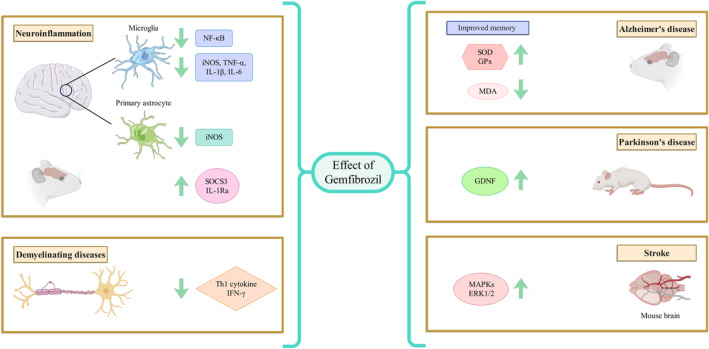
Neuroprotective effects of gem in NDs.

Gem is a drug that is used to treat high cholesterol and triglyceride levels in the blood. It belongs to a class of drugs called fibrates and works by reducing the production of triglycerides and increasing the production of HDL cholesterol. It has been found to have potential therapeutic effects in NDs. The results of this study show that Gem has neuroprotective effects through several cellular and molecular mechanisms such as: (1) Gem affects mitochondrial pro‐survival factors in the brain by modulating the expression of PGC‐1α, TFAM, and NRF‐1, (2) Gem inhibits the activation of transcription factors NF‐κB, AP‐1, and C/EBPβ, which are involved in the expression of iNOS, (3) Gem stimulates the transcription of GDNF in astrocytes via PPAR‐α, (4) Gem administration reduces amyloid plaque pathology, reduces glial activation, and improves memory, (5) Gem stimulates PPAR‐β mediated expression of myelin genes (MBP and CNPase) and (6) Gem promotes BDNF in mice, causing antidepressant effects. Figure 1 shows the neuroprotective effects of Gem in NDs (with emphasis on cellular and molecular mechanisms).

Overall, these findings suggest that Gem may have potential therapeutic benefits in neuroinflammatory and NDs by suppressing microglial activation and inflammation. However, further research is needed to fully understand the mechanisms and potential clinical applications of Gem in NDs.

## AUTHOR CONTRIBUTIONS

Mohammad Yasin Zamanian, Mehraveh Sadeghi Ivraghi, and Elham Hakimizadeh: conception, design, writing, and revising the manuscript. Elham Hakimizadeh: revising and editing the manuscript and graphic drawing. Reena Gupta, Hashem O. Alsaab, Ahmed Hjazi, and Rosario Mireya Romero‐Parra: data gathering and editing the manuscript. E.R.A, H.A and B.M.H contributed to data collection, drafting of the manuscript, and table creation. All authors read and approved the final manuscript.

## FUNDING INFORMATION

None.

## CONFLICT OF INTEREST STATEMENT

The authors declare no conflict of interest.

## Data Availability

The data relevant to the review article is within the manuscript.
